# Gait Variability and Cognitive Function in Older Adults with Unsteady Gait

**DOI:** 10.1093/geroni/igaf122.3360

**Published:** 2025-12-31

**Authors:** Juhi Digvijay Salecha, Pei-An Lee, Brad Manor, On-Yee Lo

**Affiliations:** Hebrew SeniorLife & Harvard Medical School, Boston, Massachusetts, United States; Hebrew SeniorLife & Harvard Medical School, Boston, Massachusetts, United States; Hebrew SeniorLife/Harvard Medical School, Boston, Massachusetts, United States; Harvard Medical School/ Marcus, Hebrew SeniorLife, Boston, Massachusetts, United States

## Abstract

Older adults with unsteady gait are at high risk of falls and cognitive decline. However, the cognitive mechanisms underlying unsteady gait patterns remain poorly understood. While previous research has linked gait and cognition in older adults, few studies specifically examined such relationships in individuals with pre-existing unsteady gait. This study examined the relationship between gait variability, defined by the coefficient of variation in stride time, and cognition in older adults with unsteady gait. Twenty-eight community-dwelling older adults with unsteady gait, defined by gait variability ≥0.025 (79±7 years; 19 females; BMI: 29±5, Fall Efficacy Scale: 27±8) completed walking under two conditions: walking quietly and while completing a verbalized serial subtraction dual task. Participants also completed a cognitive assessment using the NIH Toolbox, measuring processing speed (Pattern Comparison Processing Speed), executive function (Flanker Inhibitory Control and Attention), episodic memory (Picture Sequence Memory), and working memory (List Sorting Working Memory). Multiple linear regression models, adjusted for age, sex, and BMI revealed that processing speed was correlated with gait variability specifically within the dual-task condition (Adjusted R2=0.41, β=-0.50, p = 0.006). No other cognitive outcomes were correlated with gait variability in either walking conditions. These results suggest that in older adults with unsteady gait, processing speed may play a critical role in locomotor control, especially when walking is challenged by a cognitive dual task. Future studies are needed to validate these observations and to examine the extent to which processing speed and dual-task performance can be modified in this population.

